# Frequency of Zinc Deficiency Among Thalassemia Major Patients: A Comparative Cross-Sectional Study

**DOI:** 10.7759/cureus.96523

**Published:** 2025-11-10

**Authors:** Uzma Hayat, Uzma Zaidi, Saima Munzir, Shabnam Dildar, Zainab Sharif, Tasneem Farzana, Muhammad Nizamuddin, Tahir Shamsi

**Affiliations:** 1 Paediatric Haematology, Oncology and Bone Marrow Transplantation, National Institute of Blood Diseases and Bone Marrow Transplantation, Karachi, PAK; 2 Bone Marrow Transplantation, National Institute of Blood Diseases and Bone Marrow Transplantation, Karachi, PAK; 3 Haematology, National Institute of Blood Diseases and Bone Marrow Transplantation, Karachi, PAK; 4 Chemical Pathology, National Institute of Blood Diseases and Bone Marrow Transplantation, Karachi, PAK; 5 Clinical Haematology, National Institute of Blood Diseases and Bone Marrow Transplantation, Lahore, PAK; 6 Research and Development, National Institute of Blood Diseases and Bone Marrow Transplantation, Karachi, PAK

**Keywords:** beta thalassemia major, blood transfusion, hydroxyurea, iron chelator, zinc deficiency

## Abstract

Introduction: Beta thalassemia major is a common autosomal recessive blood disorder that arises due to reduced or absent synthesis of the globin chain of hemoglobin. It serves as an alternative therapy in transfusion-dependent beta thalassemia to reduce the burden of blood transfusions. The primary objective of this study was to ascertain the frequency of zinc deficiency among two distinct populations of thalassemic children: those with transfusion-dependent thalassemia and those on fetal hemoglobin (HbF) augmentation therapy (hydroxyurea). The secondary objective of this study was to evaluate the effect of hydroxyurea on zinc levels in patients receiving this drug therapy.

Methods: A comparative cross-sectional study was conducted over a period of two months following approval from the Institutional Review Board. Thalassemic patients were divided into two groups for analysis: regularly transfused patients and those on HbF augmentation therapy (hydroxyurea) with infrequent or no blood transfusions.

Results: A total of 100 patients were enrolled in this study, with a mean age of 7.7 ± 3.2 years, of which 51% were male and 49% were female. The frequency of zinc deficiency among individuals receiving regular transfusions was 56%, while the frequency of zinc deficiency among individuals taking hydroxyurea with infrequent transfusions was 42%. Although the frequency of zinc deficiency was higher among patients receiving blood transfusions, it was not statistically significant (p = 0.161).

Conclusion: In this study, it was found that hydroxyurea-treated thalassemic children had a lower frequency of zinc deficiency compared to transfusion-dependent thalassemic children; however, this difference was not statistically significant.

## Introduction

Beta thalassemia is an autosomal recessive blood disorder resulting from a quantitative defect in beta globin chain production. With a carrier incidence of 5% to 8%, it is the most common genetically transmitted blood disorder. In Pakistan, approximately 5,000 children are diagnosed with beta thalassemia annually [1]. Blood transfusion remains its most common therapeutic option.

After iron, zinc is the most common trace element necessary for growth and immune function [2]. Zinc inadequacy in patients with thalassemia may be due to defective absorption or low intake. Chelation is known to cause zinc deficiency in patients with beta thalassemia by reducing intestinal absorption. Iron chelators have been shown to decrease zinc levels in patients with beta thalassemia through a mechanism involving reduced absorption [3].

Hydroxyurea is one of the first medications that showed potential for treating hemoglobinopathies such as sickle cell disease and β-thalassemia. It reversibly inhibits the enzyme ribonucleoside diphosphate reductase, which catalyzes a crucial stage in DNA synthesis, thereby stimulating the expression of γ-globin, a fetal globin gene that is repressed after birth, in human erythroid cells. For sickle cell disease and non-transfusion-dependent beta thalassemia, hydroxyurea is now used universally to augment fetal hemoglobin (HbF). Numerous clinical trials are currently in progress to assess its efficacy in treating transfusion-dependent β-thalassemia [1-4].

Serum zinc levels have reportedly been found to be lower in beta thalassemia patients on hydroxyurea therapy than in normal control groups [5]. According to a Pakistani study by Sultan et al., among transfusion-dependent beta thalassemia patients, the frequency of zinc deficiency (<50 μg/dL) was 22%. However, it was found that 68% of children with thalassemia in Southeast Asia had zinc deficiencies [6].

Thalassemic patients are at risk of zinc deficiency due to diverse causes. Studies have demonstrated changes in the fatty acid profile of beta thalassemic patients before and after receiving hydroxyurea therapy [6,7].

This study compares the frequency of zinc deficiency between two populations of thalassemia major patients: those who are transfusion-dependent and those taking hydroxyurea with infrequent blood transfusions. The study also observes the association between hydroxyurea use and serum zinc levels, as previous studies suggest positive effects of hydroxyurea on metabolism.

## Materials and methods

A comparative cross-sectional study was conducted over a period of two months after approval from the Institutional Review Board under the Declaration of Helsinki.

Since no similar study existed on the frequency of zinc levels in thalassemic children taking hydroxyurea, we performed a pilot study that showed 42% and 56% prevalence of zinc deficiency in thalassemic children taking hydroxyurea and in the transfusion-dependent thalassemia group, respectively. Using these two frequencies, at a power of 80% and with a 95% confidence interval, a sample size of 49 per group was calculated. Therefore, we enrolled a total of 50 patients in each group. Sample size calculation was performed using the WHO sample size calculator. The sampling technique was nonprobability purposive sampling.

Thalassemic patients were divided into two groups for analysis: regularly transfused patients and those on HbF augmentation therapy (hydroxyurea) with infrequent blood transfusions.

The inclusion criteria included age between 1 and 16 years, confirmed diagnosis of beta thalassemia major (by hemoglobin electrophoresis), transfusion dependence with compliance to regular follow-up, and patients taking hydroxyurea with infrequent transfusions (need for transfusion beyond three months). Patients with normal liver and kidney function tests were included. Individuals younger than one year or older than 16 years, or those with liver disease, kidney disease, gastrointestinal disease, or specific dietary habits such as a vegetarian regimen with zinc and mineral supplementation, were excluded from the study.

Three to five milliliters of blood were collected in a gel vacutainer from each subject. Blood samples were centrifuged at 3,000 rpm for 15 minutes, aliquoted with appropriate subject identification numbers, and stored at −80°C until further analysis. Patient identity was removed to ensure confidentiality, and codes were assigned.

The analysis was performed on a Selectra ProM routine clinical chemistry analyzer (ELITechGroup SAS, Puteaux, France) using the colorimetric method. The zinc present in the sample was chelated by 5-Br-PAPS (2-(5-bromo-2-pyridylazo)-5-(N-propyl-N-sulfopropylamino) phenol) in the reagent. The formation of this complex was measured at a wavelength of 560 nm. Two-level quality control materials were run with each batch of samples. Serum zinc levels of 65-150 μg/dL were considered optimal, with levels below 65 μg/dL indicating zinc deficiency and levels above 150 μg/dL considered elevated.

The collected data were entered into IBM SPSS Statistics for Windows, Version 26 (Released 2019; IBM Corp., Armonk, New York). Categorical variables were expressed as frequency and percentage, and numerical variables were expressed as mean ± standard deviation. Qualitative data were compared between the two study groups using the chi-square test. Numerical data were compared using the Mann-Whitney U test, as they were not normally distributed. A p-value less than or equal to 0.05 was considered statistically significant.

## Results

A total of 100 patients were included in the study, with a mean age of 7.7 ± 3.2 years (IQR, 5-8 years), and almost equal male (51%) and female participation (49%). The average heights and weights of patients were 115.5 ± 17.5 cm and 20.7 ± 7.1 kg, respectively. Twenty-three percent had a weight ≤5th percentile, 53% were up to the 10th percentile, and 24% were above the 50th percentile. Half of the patients in this study were transfusion-dependent, whereas the remaining were receiving hydroxyurea therapy with infrequent blood transfusions.

**Figure 1:**
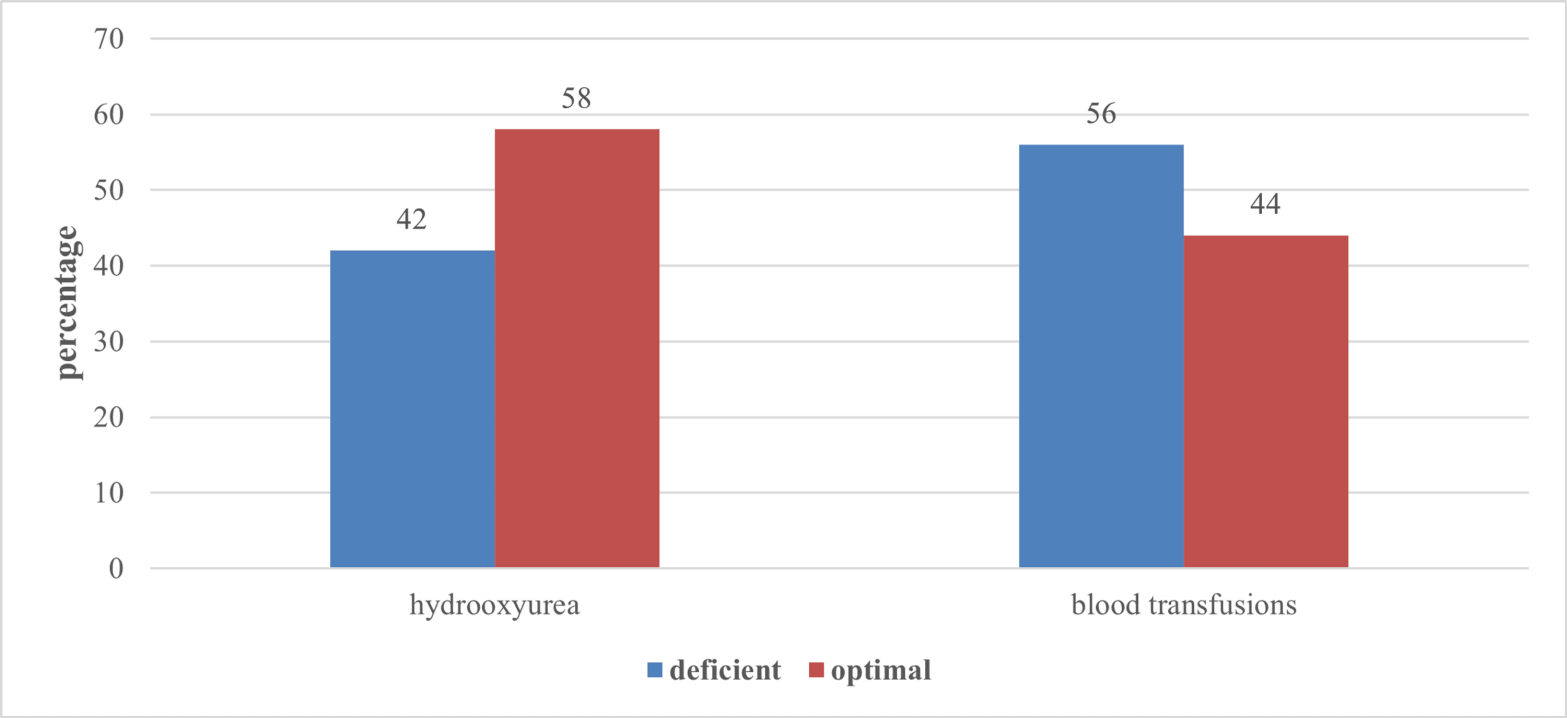
Frequency of zinc deficiency among patients receiving hydrooxyurea and blood transfusion

A palpable spleen was observed in one-third of all patients (33%). Other clinical features included insomnia (7%), frequent diarrhea (4%), repeated flu onset (2%), loss of appetite (3%), difficulty in wound healing (1%), malabsorption (2%), allergy (1%), irritability (31%), difficulty in learning (5%), and behavioral problems (9%). None of the patients had hair loss or loss of smell or taste. Median levels of ferritin and alanine aminotransferase were 2,200 (IQR, 1,612.5-3,181.7) and 21 (IQR, 12-27), respectively. Table 1 presents the sociodemographic and clinical profiles of the two study populations. Figure 1 presents the frequency of zinc deficiency and optimal zinc levels among the study groups. The frequency of zinc deficiency among transfusion-dependent thalassemia major patients was 56%, and the frequency among patients taking hydroxyurea with infrequent transfusions was 42%. Elevated zinc levels (>150 μg/dL) were not observed in this study. Although the frequency of zinc deficiency was higher among patients receiving blood transfusions, it was not statistically significant (p = 0.161).

**Table 1:** A comparison of sociodemographic and clinical profile of patients who received and did not receive hydrooxyurea P-values were measured using the chi-square test. **Mann-Whitney U-test or T-test. ^†^Fisher exact test. SGPT: serum glutamate pyruvate transaminase.

Variables	Groups	With hydroxyurea	With blood transfusions	Test statistic	p-value
Age (in years)	-	9 (6.7-11)	6 (5-8)	25.752	0.001**
Gender	Male	25(49)	26(51)	1.287	0.841
	Female	25(51)	24(49)		
Percentile	5.00	22(95.7)	1(4.3)	5.531	0.054
	10.00	19(35.8)	34(64.2)		
	50.00	9(37.5)	15(62.5)		
Insomnia	Yes	7(100)	0(0)	10.473	0.012^†^
	No	43(46.2)	50(53.8)		
Frequent diarrhea	Yes	4(100)	0(0)	2.347	0.117^†^
	No	46(47.9)	50(52.1)		
Repeated onset of flu	Yes	2(100)	0(0)	1.874	0.495^†^
	No	48(49)	50(51)		
Loss of appetite	Yes	3(100)	0(0)	1.572	0.242^†^
	No	47(48.5)	50(51.5)		
Difficulty in healing wounds	Yes	1(100)	0(0)	0.478	1.000^†^
	No	49(49.5)	50(50.5)		
Malabsorption	Yes	2(100)	0(0)	1.874	0.495^†^
	No	48(49)	50(51)		
Allergy	Yes	1(100)	0(0)	0.478	1.000^†^
	No	49(49.5)	50(50.5)		
Irritability	Yes	18(58.1)	13(41.9)	1.471	0.280
	No	32(46.4)	37(53.6)		
Learning difficulty	Yes	5(100)	0(0)	5.147	0.056^†^
	No	45(47.4)	50(52.6)		
Behavioral problems	Yes	9(100)	0(0)	20.471	0.003^†^
	No	41(45.1)	50(54.9)		
Palpable spleen	Yes	23(69.7)	10(30.3)	19.754	0.004**
	No	26(39.4)	40(60.6)		
Ferritin	-	2460.5 (1800-3420)	2100 (1600-2641.2)	10.745	0.018**
Oral chelators	Single	40 (80)	46 (92)	4.143	0.084
	Combination	10 (20)	4 (8)		
SGPT	-	21 (12-33)	20 (11.7-23.3)	4.177	0.084**

Table 2 presents the comparison of serum zinc deficiency between the hydroxyurea and transfusion-dependent groups, as well as the comparison of zinc deficiency between the two study groups after stratification by gender, weight percentile, ferritin levels, alanine aminotransferase levels, and palpable spleen. Zinc deficiency was not significantly different between the two study populations, even after stratification by gender, weight percentile, ferritin level, serum glutamate pyruvate transaminase (SGPT) level, palpable spleen, and use of chelators.

**Table 2:** A comparison of zinc deficiency among study groups P-values were measured using the chi-square test. ^†^Fisher exact test. SGPT: serum glutamate pyruvate transaminase.

Variables	Groups	Zinc status	Hydrooxyurea, n(%) (group 1)	Blood transfusion, n(%) (group 2)	Test statistic	p-value
Gender	Male	Deficient	14(56)	12(46.2)	1.147	0.482
		Optimal	11(44)	14(53.8)		
	Female	Deficient	14(56)	9(37.5)	1.345	0.195
		Optimal	11(44)	15(62.5)		
Weight percentile	3rd percentile	Deficient	14(63.6)	13(38.2)	1.478	0.168
		Optimal	8(36.4)	21(61.8)		
	10th percentile	Deficient	11(57.9)	1(100)	0.589	1.000
		Optimal	8(42.1)	0(0)		
	50th percentile	Deficient	3(33.3)	7(46.7)	1.014	0.678^†^
		Optimal	6(66.7)	8(53.3)		
Ferritin levels	≤2200	Deficient	10(47.6)	11(35.5)	1.247	0.382
		Optimal	11(52.4)	20(64.5)		
	>2200	Deficient	18(62.1)	10(52.6)	1.357	0.517
		Optimal	11(37.9)	9(47.4)		
SGPT levels	≤21	Deficient	14(53.8)	15(50)	1.040	0.774
		Optimal	12(46.2)	15(50)		
	>21	Deficient	14(58.3)	6(30)	2.573	0.060
		Optimal	10(41.7)	14(70)		
Palpable spleen	Yes	Deficient	13(56.5)	6(60)	0.478	1.000^†^
		Optimal	10(43.5)	4(40)		
	No	Deficient	14(53.8)	15(37.5)	1.578	0.191
		Optimal	12(46.2)	25(62.5)		
Chelators	Single	Deficient	17(42.5)	26(56.5)	1564	0.195
		Optimal	23(57.5)	20(43.5)		
	Combination	Deficient	4(40)	2(50)	0.578	1.000^†^
		Optimal	6(60)	2(50)		

## Discussion

Blood transfusion and iron chelation remain the primary therapeutic modalities for the majority of thalassemia patients in low- and middle-income countries, where access to hematopoietic stem cell transplantation is extremely limited. However, these treatments are associated with significant drawbacks, including iron overload, transfusion-transmitted infections, organ dysfunction, and deficiencies in essential micronutrients such as zinc, all of which contribute to a diminished quality of life for affected individuals. Hydroxyurea, one of the most commonly used pharmacologic agents for augmenting HbF, enhances γ-globin chain synthesis and has shown potential in mitigating disease severity [7].

Thalassemic patients are particularly susceptible to zinc deficiency due to multiple contributing factors, including chelation therapy with desferoxamine, inadequate dietary intake, and increased urinary excretion of zinc. A study conducted in Iran by Dehsal reported a zinc deficiency prevalence of 37% among patients with thalassemia major. Similarly, Ferdaus et al. found that 60% of Bangladeshi thalassemic children exhibited low serum zinc levels [8]. In our study, 56% of thalassemic children receiving only blood transfusions were zinc-deficient, compared to 42% of those on hydroxyurea therapy. While previous research indicates that zinc levels in hydroxyurea-treated children are lower than in the general population [5-9], our findings suggest a relatively lower frequency of zinc deficiency in this group compared to transfusion-dependent patients. However, this observed difference was not statistically significant.

This trend may be explained by the fact that children on hydroxyurea often require fewer transfusions, resulting in comparatively reduced iron burden and less aggressive chelation therapy, both of which contribute to lower urinary zinc loss. In contrast, transfusion-dependent patients are more likely to receive intensive iron chelation, especially desferoxamine or deferasirox, which are known to cause hyperzincuria and decreased plasma zinc levels. Additionally, the chronic oxidative stress induced by iron overload may further interfere with zinc metabolism in transfusion-dependent patients.

Growth impairment is a well-documented complication in thalassemic children, with reported global prevalence ranging between 25% and 60%. Despite notable therapeutic advances, growth failure remains a considerable challenge impacting the quality of life [9]. In the present study, 68.6% of patients receiving hydroxyurea were found to be underweight and stunted, falling below the third percentile. In contrast, 38.2% of transfusion-dependent patients were underweight. These findings contrast with those reported by Moiz et al., who found that 65% of patients were stunted and 40% were underweight [10].

The greater proportion of growth retardation in our hydroxyurea group may be attributed to low hemoglobin levels that hinder normal growth. In the transfusion-dependent group, the optimal goal was to maintain hemoglobin above 9 g/dL. Good hemoglobin levels support organ development and overall physical and mental well-being. Furthermore, hydroxyurea-treated patients often come from more socioeconomically disadvantaged groups who may opt for oral therapy over transfusions due to cost or accessibility, thus compounding nutritional deficiencies and growth failure. It is also possible that in our study setting, transfusion-dependent patients were under closer clinical supervision, receiving more regular anthropometric monitoring and dietary guidance, contributing to comparatively better growth parameters.

Previous literature presents mixed evidence regarding the correlation between zinc levels and growth parameters. El Missiry et al. (2014) found no significant relationship between serum zinc concentrations and growth indices among regularly transfused thalassemic children [11]. Karunaratna et al. also observed that 50% of patients were stunted, attributing growth retardation to chronic anemia, long-term chelation therapy, and socioeconomic factors [12]. The multifactorial nature of growth retardation in thalassemia is widely acknowledged and includes contributors such as iron toxicity, chronic hypoxia, and nutritional deficiencies.

An Italian study demonstrated that 18% of thalassemic patients had growth parameters below the fifth percentile, suggesting that therapeutic interventions may be inadequate or suboptimal [13]. Previous studies have shown that excessive iron accumulation from repeated transfusions, along with ineffective erythropoiesis, hinders zinc absorption in the gastrointestinal tract. An inverse relationship between ferritin and serum zinc levels has been documented, with higher ferritin concentrations correlating with reduced serum zinc levels. Studies by Arijanty et al. and Nima et al. support this negative correlation between plasma zinc and serum ferritin levels.

Similar conclusions were drawn by Mahyar et al. and El Missiry et al., although the latter reported this correlation as statistically insignificant [11-14]. Arijanty (2006) also reported no significant differences in zinc levels between thalassemic children and healthy controls, nor any meaningful correlation between serum ferritin and zinc concentrations [15-16]. Nonetheless, our study findings suggest that elevated ferritin levels are associated with lower serum zinc concentrations.

This relationship may be explained by the fact that iron and zinc share common absorption pathways in the gut, particularly via divalent metal transporter 1 (DMT1). As iron stores increase, zinc absorption is competitively inhibited, leading to functional zinc deficiency. Moreover, the inflammatory milieu associated with iron overload may increase hepatic metallothionein expression, which sequesters zinc and reduces its bioavailability. These mechanistic links may account for the inverse pattern observed between serum ferritin and zinc concentrations.

Zinc deficiency in beta thalassemia patients has also been linked to the use of both intravenous and oral iron chelators, which can induce hyperzincuria, with an estimated prevalence of 37%. Sultan et al. reported hypozincemia among thalassemic children undergoing regular transfusions and receiving intravenous desferoxamine [6]. Erdogan et al. found that serum zinc levels were lower among transfusion-dependent patients treated with oral chelators such as deferiprone and deferasirox [8-17]. Chronic zinc deficiency in thalassemia has been attributed to altered zinc-binding capacity due to long-term treatment and disease pathology [16].

Munira et al. observed that mean serum zinc concentrations were significantly lower in transfusion-dependent thalassemia patients compared to healthy controls [18]. The implications of zinc deficiency are considerable, with studies such as that by Suzan linking reduced zinc levels to insulin resistance. Specifically, serum zinc was significantly lower in patients with serum ferritin above 2,500 ng/mL, a finding associated with impaired insulin secretion and an increased risk of diabetes mellitus [19].

Mahyar et al. further reported that hypozincemia is prevalent among patients with thalassemia major and identified several causative factors, including low dietary intake, renal dysfunction, increased urinary zinc excretion, disruptions in zinc metabolism, and elevated sweat losses [14-20]. Tabatabaei et al. documented zinc deficiency in 84.8% of thalassemia major patients [21], while Yazdiha et al. demonstrated significantly lower serum zinc levels in thalassemic patients compared to controls and advocated for zinc supplementation in this population [22]. Conversely, Reshadat et al. found no reduction in plasma zinc levels among thalassemia major patients [23]. Modaresi et al. observed reduced zinc concentrations in hair samples from thalassemia major patients, attributing the deficiency to malnutrition and insufficient dietary intake [24]. Fahim et al. revealed that 49% of patients experienced short stature and 47% were underweight, with primary causes identified as iron overload, chelation therapy, and inadequate nutritional support [25].

In our study, zinc deficiency was directly related to patients taking oral chelators, with a significant p-value. Approximately 42.5% and 56.5% of patients taking single oral chelators were deficient in serum zinc levels in group 1 and group 2, respectively, whereas 40% and 50% of patients taking a combination of chelators were deficient in serum zinc levels in group 1 and group 2, respectively.

In this study, we evaluated the frequency of zinc deficiency among thalassemic children: those entirely dependent on blood transfusions and those taking hydroxyurea with infrequent transfusions. We also assessed the growth and development of children with thalassemia.

Zinc is one of the most essential minerals required for multiple body functions and plays a vital role in promoting the growth and development of children, including those with thalassemia. Hydroxyurea, a revolutionary drug, has tremendously impacted the health of thalassemic children. It has also shown beneficial effects on several metabolic functions, including maintaining the zinc levels of thalassemic children. This study highlights that zinc levels in thalassemic children taking hydroxyurea show decreased zinc deficiency compared to thalassemic children solely on blood transfusion.

Strengths

The prevalence of zinc deficiency in thalassemic children taking hydroxyurea has been documented in this study. Notably, the prevalence of zinc deficiency in transfusion-dependent thalassemic children was higher compared to thalassemic children taking hydroxyurea, although the difference was not statistically significant.

Limitations

This is a single-center study conducted on a small scale. The dietary regimen of the children could not be assessed, as they were not observed in person.

## Conclusions

In this study, children receiving hydroxyurea therapy demonstrated a comparatively lower frequency of zinc deficiency; however, the difference between the treatment and control groups was not statistically significant. In patients with thalassemia, serum zinc levels should be evaluated every three months to rule out zinc deficiency and to ensure optimal growth through zinc supplementation when deficiency is documented.

Nevertheless, in children with thalassemia, growth impairment is multifactorial and cannot be attributed solely to zinc status. The underlying disease process, along with complications such as chronic hypoxia, iron overload, and the use of iron chelators, exerts a cumulative negative impact on linear growth and overall development. These confounding factors may mask the potential benefits of improved zinc status, thereby limiting the observable effects of interventions such as hydroxyurea on growth outcomes in this population.
